# Tumor Necrosis Factor-Alpha: Ally and Enemy in Protean Cutaneous Sceneries

**DOI:** 10.3390/ijms25147762

**Published:** 2024-07-16

**Authors:** Krizia Pocino, Valeria Carnazzo, Annunziata Stefanile, Valerio Basile, Cristina Guerriero, Mariapaola Marino, Donato Rigante, Umberto Basile

**Affiliations:** 1 Unità Operativa Complessa di Patologia Clinica, Ospedale San Pietro Fatebenefratelli, 00189 Rome, Italy; pocino.krizia@fbfrm.it (K.P.); stefanile.nunzia@gmail.com (A.S.); 2 Department of Clinical Pathology, Santa Maria Goretti Hospital, 04100 Latina, Italy; v.carnazzo@ausl.latina.it (V.C.); u.basile@ausl.latina.it (U.B.); 3 Clinical Pathology Unit and Cancer Biobank, Department of Research and Advanced Technologies, Regina Elena National Cancer Institute IRCCS, 00144 Rome, Italy; valeriobasile90@gmail.com; 4 Department of Dermatology, Fondazione Policlinico Universitario A. Gemelli IRCCS, 00168 Rome, Italy; cristina.guerriero@policlinicogemelli.it; 5 Dipartimento di Medicina e Chirurgia Traslazionale, Università Cattolica Sacro Cuore, 00168 Rome, Italy; 6 Fondazione Policlinico Universitario A. Gemelli IRCCS, 00168 Rome, Italy; donato.rigante@unicatt.it; 7 Department of Life Sciences and Public Health, Università Cattolica Sacro Cuore, 00168 Rome, Italy

**Keywords:** tumor necrosis factor-alpha, skin, inflammation, innovative biotechnologies, precision medicine

## Abstract

Skin is the forestage for a series of many-sided functions of tumor necrosis factor-alpha (TNF-α), a proinflammatory cytokine with staggering versatility and sizable implications for tissue homeostasis, immune responses, angiogenesis, apoptosis, local and systemic inflammation. An aberrant TNF-α-mediated crosstalk has been linked to the pathogenesis of acute and chronic skin inflammatory diseases, and indeed, TNF-α dysregulation can contribute to the development and progression of psoriasis, vitiligo, local damage following exposition to ultraviolet light radiations, cutaneous lupus erythematosus, and acne vulgaris. Therapies that target TNF-α are conspicuously used in the treatment of different skin disorders, aiming to modulate the in vivo immune functions triggered by many cutaneous cells, including keratinocytes, mast cells, or Langerhans cells, and reduce inflammation taking place within the skin. Herein, we focus on the key relationships between TNF-α and distinct skin non-neoplastic inflammatory or physiologic conditions, showing that a natural induction of TNF-α may have a protective significance but that TNF-α overproduction may be harmful or even lethal. Many questions remain unraveled in the therapeutic practice, and caution should be exercised due to eventual backlashes exerted by TNF-α in maintaining skin health or in provoking skin disease.

## 1. Introduction

**Figure 1:**
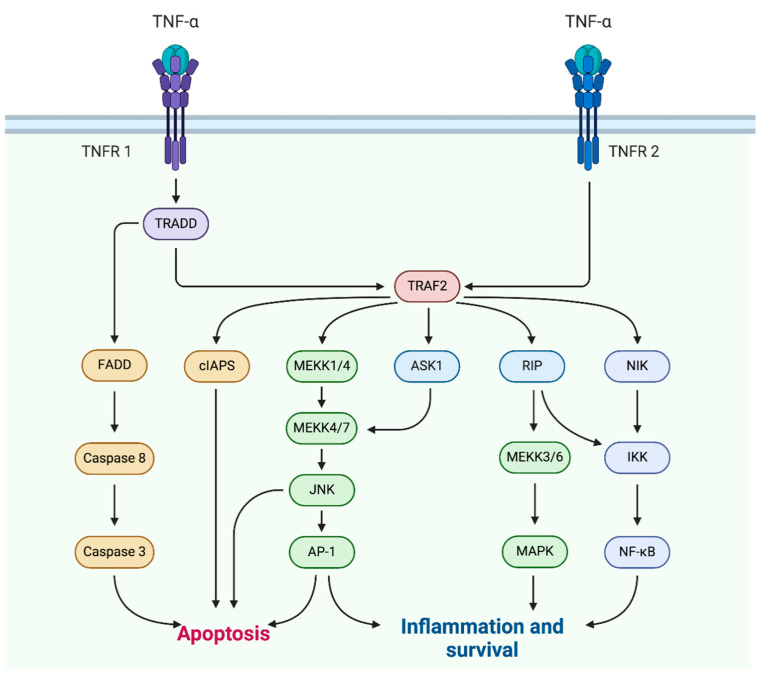
Tumor necrosis factor-alpha (TNF-α) signaling pathways: TNF-α binds to the transmembrane receptors TNFR1 or TNFR2, leading to apoptosis or starting inflammation. The activation of TNFR1 leads to the formation of a death-inducing signaling complex containing TRADD, FADD, and caspase 8: this complex triggers the induction of apoptosis, culminating in the cleavage of caspase 3. Proinflammatory and survival signaling pathways can also be induced by the activation of TNFR1 or TNFR2 through the adapter protein TRAF2, which in turn activates RIP1, NIK, or MEK, resulting in the activation of MAPK and NF-kB.

Tumor necrosis factor-alpha (TNF-α) was discovered in 1975 as a molecule released into the bloodstream after reticuloendothelial system stimulation with bacterial lipopolysaccharide and was originally characterized as an anti-tumor and cytotoxic agent for many malignant cells, as it was capable of inducing the necrotic regression of certain tumors [1]. Understanding the role of TNF-α, also known as cachectin, in the skin layers is pivotal for unraveling the protean mechanisms underlying a host of cutaneous disorders, which would require the development of more targeted and rescissory therapeutic interventions. It is crucial to admit that TNF-α regulation at the skin level is convolute and that its effects may vary depending on the specific context and pathologic sceneries. Firstly, TNF-α is a strong proinflammatory cytokine, physiologically produced by various cell types, including immune cells like macrophages, T lymphocytes, and natural killer cells, largely involved in either physiological or disease processes, comprising the response to invading microorganisms and subsequent inflammation within the invaded district [2]. Bacterial lipopolysaccharide has long been considered one of the main triggers of TNF-α production [3], but also viral, mycotic and parasitical antigens as well as enterotoxins, complement proteins, superantigens, immune complexes, interleukin (IL)-1, interferon (IFN), granulocyte-macrophage colony-stimulating factor, transforming growth factor- α, and TNF-α itself—through autocrine mechanisms—have the power to induce the release of TNF-α [4]. Conversely, TNF-α biosynthesis is weakened by IL-4 or other agents that decrease the level of cyclic adenosine monophosphate [5]. Additionally, it has been demonstrated that cells do not store TNF-α, though they can swiftly induce a de novo synthesis of this cytokine under different stimulating promoters [6]. Moreover, TNF-α itself may stimulate the release of anti-inflammatory factors, such as IL-10, endogenous corticosteroids, and prostanoids, that are capable of negatively regulating and counterbalancing its expression [4]. On the whole, the network orchestrated by TNF-α is crucial for the communication between resident cells of different districts and inflammatory cells, like neutrophils, lymphocytes, mast cells, or Langerhans cells, contributing to control of the progress of inflammation and also inhibit its extent and duration [7]. Several intracellular signaling events are exerted by TNF-α through its binding to cell membrane-bound TNF-receptor (TNFR)1 and TNFR2: both of them are transmembrane glycoproteins, but TNFR1 is highly promiscuous and expressed on every cell type in the body, while TNFR2 is limited to cells of the immune system, endothelial and nerve cells [8]. Furthermore, the TNFR1-associated death domain (TRADD), an adapter molecule that bridges the interaction with different kinases, may be responsible for cell apoptosis, following caspase-8 oligomerization and activation via Fas-associated death domain (FADD); caspase-8 then activates downstream effectors, committing the involved cell to apoptosis [8]. In the context of skin, TNF-α plays a significant role in the regulation of tissue homeostasis, cell survival, and inflammation, inducing the production of adhesion molecules and chemokines, but also promoting the attachment of inflammatory cells to vessels, their rolling and chemotaxis [4]. The activation of immune cells, such as neutrophils and macrophages, operated by TNF-α is aimed to eliminate potential pathogens and warrant a pathogen-specific immune response [9]. A further role of TNF-α is the induction of apoptosis in keratinocytes, the predominant cell type in epidermis [10]. This process is part of the normal physiologic turnover of skin cells and is important for removing either infected or damaged cells, contributing to immune surveillance and maintaining the functional integrity of the skin [10]. In addition, TNF-α fosters the shedding of dead cells and proliferation of new ones: a dysregulation of this process may result in abnormal skin cell turnover and hyperproliferation of keratinocytes, leading to conditions characterized by scaling and thickening of the skin, as well-known for psoriasis (Figure 1).

## 2. TNF-α in Skin Diseases

### 2.1. Psoriasis

The role of TNF-α is overriding in the pathophysiology of psoriasis, a chronic skin disorder characterized by aberrant immune system activation followed by accelerated cell turnover, resulting in the formation of thick, scaly, and red patches on the skin [2]. Psoriatic lesions typically exhibit increased levels of TNF-α [11], which trigger an inflammatory cascade culminating in tissue damage via recruitment of immune cells to the skin plaques [2,12]. However, TNF-α works in concert with other cytokines, such as IL-17 and IL-23, sustaining the inflammatory response at the epidermis level [2]. By synergizing with TNF-α, IL-17A induces the release of chemokine C-C-motif ligand (CCL)20, which stimulates the recruitment of IL-17-producing cells, which further increases the production of inflammatory mediators [13]. In particular, together with IL-23, TNF-α promotes the differentiation of naïve T cells into Th17/Tc17 cells, which not only produce IL-17 but also activate innate lymphoid cells (ILCs). These multifunctional cells play a role in innate immunity at the barrier surface, where they promote tissue remodeling, lipid catabolism, and communication between the neuronal and immune systems. ILCs are highly plastic and have also the potential to transdifferentiate from one subset into another. In fact, TNF-α can activate ILC3 and convert ILC2 to ILC3 [14]. Notably, the accumulation of these cells within the skin lesions of patients with psoriasis reveals that ILC2-to-ILC3 plasticity would play a pathogenic role in diseases characterized by IL-17 overproduction [14].

**Figure 2:**
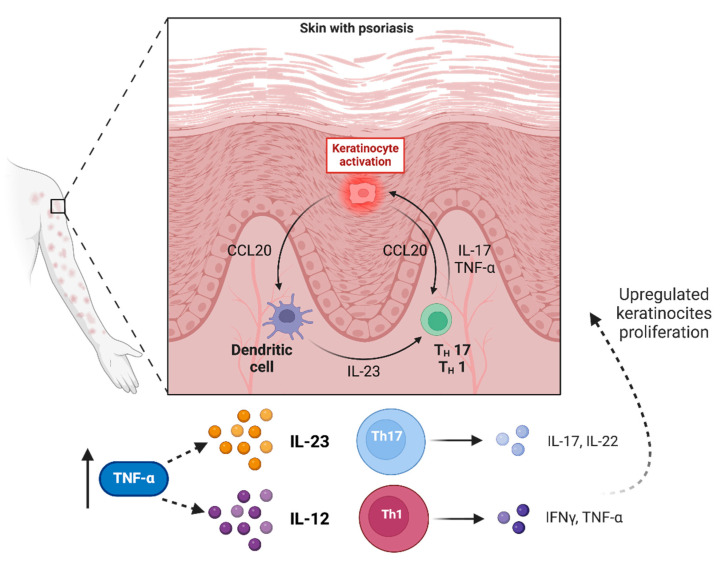
The role of keratinocytes and tumor necrosis factor-alpha (TNF-α) in the pathogenesis of psoriasis: TNF-α amplifies the inflammatory response through several distinct pathways: (a) facilitating the entry of inflammatory cells into lesional skin areas through induction of adhesion molecules on the vascular endothelial cells; (b) stimulating keratinocyte proliferation; (c) leading to the production of different proinflammatory mediators (interleukin-23, interleukin-12, interleukin-17, interleukin-22, interferon-γ) secreted by T-helper 1 and T-helper 17 cells; (d) activating dermal macrophages and dendritic cells.

TNF-α can also stimulate the production of intercellular adhesion molecule-1 (ICAM-1) by keratinocytes and endothelial cells: the interaction between endothelial cells and leukocytes is regulated by multiple receptor–ligand systems, including different adhesion molecules [15]. ICAM-1 is a ligand for lymphocyte function-associated antigen-1 (LFA-1), a cell surface antigen expressed on T lymphocytes, enabling their interaction with both keratinocytes and endothelia. Increasing the amount of adhesion molecules increases the chance of T cell binding, allowing infiltration of T lymphocytes into the skin; in addition, a direct correlation exists between endothelial cells producing ICAM-1 and areas of dermal inflammation in psoriasis [11,16]. TNF-α may even augment the synthesis of other adhesion molecules, and cells from psoriatic skin show strong staining for P-selectin and E-selectin on endothelial cells and for vascular cell adhesion molecule (VCAM)-1 on dermal fibroblasts and dendritic cells [11]. TNF-α is also capable of stimulating Langerhans cells, the antigen-presenting cells of the epidermis, to migrate from the skin to lymph nodes where T cell activation takes place: this is facilitated by decreased E-cadherin expression by TNF-α, which normally retains Langerhans cells in the epidermis [17]. TNF-α in normal skin is predominantly localized to the basal cell layer of the epidermis and largely found near eccrine ducts or sebaceous glands; in the psoriatic skin and to a lesser extent also in the uninvolved skin of patients with psoriasis, TNF-α is distributed throughout the epidermis, and also specifically on the dermal blood vessels [18] (see Figure 2).

Notably, the TNF-α gene −238G>A polymorphism (rs361525) has been related to increased incidence of psoriasis [19]. In fact, psoriatic patients have higher frequencies of the A allele and the AA genotype than healthy control groups; moreover, patients with a family history of psoriasis show an increased frequency of the AA genotype compared with GG and GA genotypes [19]. Therapies targeting both TNF-α and IL-17 or IL-23 have been specifically developed to address the complex immune dysregulation seen in psoriasis [20]. Given the central role of TNF-α, the introduction of therapies that specifically target TNF-α, including infliximab, adalimumab, etanercept, and others, have significantly transformed the overall landscape of psoriasis management. While TNF inhibitors have shown effectiveness, they could potentially give side effects, and their use requires careful monitoring over time. Indeed, individual responses to treatments may vary, and healthcare providers should consider factors such as the severity of psoriasis and potential secondary effects when choosing peculiar treatment plans.

### 2.2. Vitiligo

The expression of TNF-α is an essential key step of melanocyte dysfunction, which is disrupted in vitiligo, a chronic skin disorder characterized by loss of pigment-producing cells resulting in skin patches of depigmentation [21]. The exact pathogenesis of vitiligo is not completely understood, though it probably involves a combination of genetic, autoimmune, and environmental cues [22]. TNF-α induces alterations in melanogenesis, acting upon the microphthalmia-associated transcription factor (MITF), melanocyte-stimulating hormone receptor (MSH-R), and melanocortin-1 receptor (MC1-R), by which the expression of melanin synthase, modulating melanocyte survival and activity in normal and pathological conditions, depends on [21]. In particular, TNF-α downregulates both MITF and MSH-R function and reduces the expression of MC1-R mRNA [23].

The generation of a redox imbalance and the overproduction of reactive oxygen species (ROS) could represent other possible mechanisms of TNF-α-induced melanocyte toxicity [24]. An increased level of ROS should lower antioxidant capacity along with lipid peroxidation and multiple DNA damages, creating a pro-oxidant microenvironment that results in tissue impairment with subsequent generation of neoantigens, triggering autoimmune phenomena [25]. Both cytokine imbalance and oxidative stress in the skin crosstalk play an essential role in maintaining melanocyte homeostasis. Singh et al. performed a comparative analysis of TNF-α transcript levels from the skin of vitiligo patients (lesional and non-lesional) and controls: the lesional as well as non-lesional skin exhibited significantly higher TNF-α transcript levels as compared to control skin. The authors also found that melanocytes, upon exogenous stimulation with TNF-α, had reduced melanocyte melanin content, upregulation of TNFR1, IL-6, and ICAM1 expression, whereas TNFR2 levels were unchanged [26]. TNF-α upregulating ICAM-1 levels on the melanocyte cell surface may also enhance the T cell/melanocyte adherence to the skin, resulting in a further decrease of functional melanocytes [27]. Nevertheless, TNF-α appears also to play a protective role in vitiligo, activating and promoting the paradoxical development of T-reg cells, a subset of regulatory cells that can secrete IL-10, suppress T cell proliferation, and prevent activation of other components of the immune response [26]. 

TNF-α also plays a crucial role in the development of cytotoxic T lymphocytes implicated in the initiation of vitiligo, enhancing the release of IFN-γ, a cytokine directly involved in the depigmentation, exacerbating the local autoimmune response [28,29,30]. Serum and tissue TNF-α levels directly correlate with the vitiligo extent, duration, and activity [31,32]. In this context, TNF-α inhibitors were found to halt depigmentation, but while the activity of vitiligo can be controlled with TNF-α inhibition, real-life data do not support a correlation between disease duration prior to anti-TNF-α exposure and treatment efficacy [33]. In fact, unexpected worsening of a pre-existing vitiligo and de novo development of vitiligo lesions were observed in a minority of patients treated with anti-TNF-α agents due to other autoimmune disorders, discouraging the popularity of this strategy [34,35,36,37,38]. Knowing that TNF-α activates T-regs has led to find that depleting TNF-α can lead to decreased T-reg production, allowing cytotoxic T lymphocytes to exert an unchecked inflammatory response within the epidermis and tipping the scale in favor of depigmentation [39]. Such local T-reg deficiency might be overcome by recruiting T-regs to the skin by means of topical administration of CCL22 (C-C motif chemokine ligand 22) DNA, as demonstrated in vitiligo-prone mice [40]. A further important observation is that halting depigmentation is not necessarily followed by the promotion of repigmentation [33]. Therefore, a combination of treatments is probably the most effective tool for the optimal management of vitiligo.

### 2.3. Cutaneous Lupus Erythematosus

A chronic connective tissue disorder that causes erythema, scaling, depigmentation, and scarring of skin is cutaneous lupus erythematosus (CLE), a multifactorial autoimmune disorder with several clinical subtypes [41]. Despite a host of studies reported in the medical literature, the pathogenesis of CLE is not well deciphered, though TNF-α plays a decisive role together with environmental and genetic factors [42,43,44]. The main triggering environmental factor is skin irradiation, which may alter the morphology and function of keratinocytes, directly inducing the production of TNF-α, which also promotes vascular permeability, recruitment, and activation of macrophages and neutrophils, leading to apoptosis [45].

**Figure 3:**
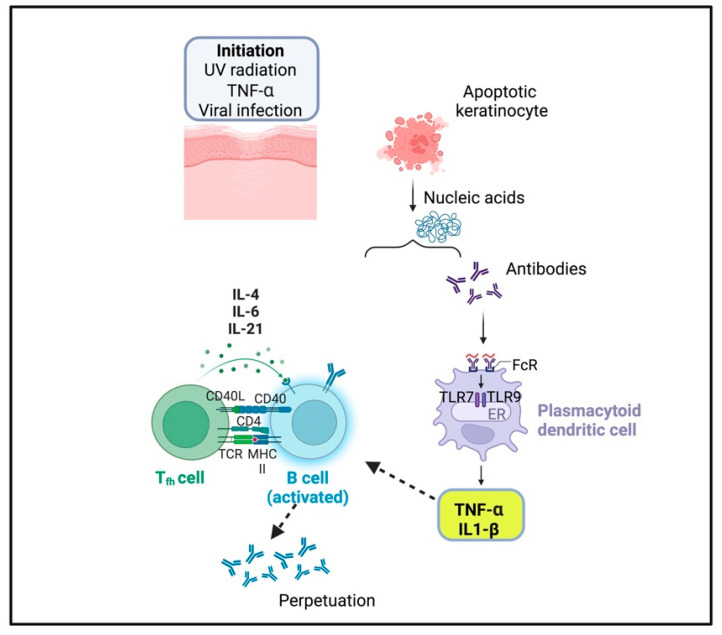
The potential effect of tumor necrosis factor-alpha (TNF-α) on keratinocyte apoptosis in the pathogenesis of cutaneous lupus erythematosus: keratinocyte apoptosis can be initiated by ultraviolet light radiations, viruses, and cytokines such as TNF-α. Apoptosis leads to the formation of small blebs that contain potential autoantigens. This process leads to the release of RNA and dsDNA, which activate TLRs within plasmacytoid dendritic cells, amplifying TNF-α, IL-1β, IL-1, IL-6, and IL-21 production. The presence of a proinflammatory environment leads antigen-presenting cells to activate the interaction between T and B cells, triggering an overproduction of autoantibodies.

Sera and skin lesions of CLE patients display a higher level of TNF-α expression [46]. TNF-α serves as a growth factor for B cells, inducing the production of IL-1, IL-6, IL-18, and IFN-γ [41]. Excessive production of these cytokines is associated with the production of autoantibodies that are deposited at the dermal-epidermal junction, causing antibody-dependent cell-mediated cytotoxicity [47]. Given the heterogeneity of this disorder, the identification of a biomarker for early diagnosis and prognosis should be important. It was reported the effectiveness of antimalarials as first-line drugs for the treatment of CLE, showing that patients treated with quinacrine (QC) had better responses than those treated with the combination of QC and hydroxychloroquine (HCQ) [48]. QC was more effective in suppressing both TNF-α and IL-6 in the peripheral blood mononuclear cells isolated from patients with CLE [48]. In skin lesions, the increase of myeloid dendritic cells with higher expression of TNF-α was predictive of poorer response to treatment with HCQ [48]. Accordingly, with this result, other studies found that approximately 50% of CLE patients were not responsive to HCQ monotherapy and that those who did not respond to HCQ were more often treated with a combination of HCQ and QC [49,50,51]. Furthermore, thalidomide, due to its anti-inflammatory properties, may work in the treatment of discoid lupus erythematosus (DLE), a subtype of CLE, inhibiting the synthesis of TNF-α [52] and leading to reduction not only of TNF-α, essential for the activation of macrophages, T and B cells, but also of IL-4, IL-5, IL-13, IL-17 or inflammatory cytokines such as IL-1, IL-2, IL-6, IL-8, IL-10, IL-12 and IFN-γ, subsequently decreasing the recruitment of immune cells to the site of injury [53]. Several case reports have shown DLE and CLE progression after treatment with TNF-α inhibitors, explaining the reasons why such drugs may not constantly be appropriate for the treatment of these conditions [54,55] (Figure 3).

Although the management of CLE by anti-TNF-α drugs is not yet well-defined, though different studies have tested their effectiveness in the most severe forms, Danielle et al. presented one case in whom the fully human monoclonal anti-TNF-α antibody adalimumab aggravated a persistent DLE [56]. A genetic study has highlighted that the TNF-α polymorphism −308A/G increases the risk and prevalence of DLE [57], although, in DLE skin lesions, gene expression microarray technique and miRNA screening have shown an enrichment of CD4+ T cells rather than CD8+ T cells, mainly promoting the production of TNF-α [58]. Molecular studies aimed at defining the regulation of the TNF promoter revealed that the −308A polymorphism might be linked to photosensitivity in patients with subacute forms of systemic lupus erythematosus [59].

### 2.4. Acne Vulgaris and Acne Inversa

Acne vulgaris is a chronic inflammatory disease of the pilosebaceous unit, mainly localized on the face, chest, and shoulders [60]: its pathogenesis is mind-bending and probably involves the species *Cutibacterium acnes*, which stimulates inflammatory and immune responses through a variety of mechanisms as the release of lipases, proteases (that destroy the hair follicle wall), and chemokines recruiting CD4+ lymphocytes, neutrophils, and monocytes to the affected site [60,61]. A significantly increased production of cytokines, including TNF-α, IL-1β, and granulocyte-macrophage colony-stimulating factor, characterizes this disorder with increasing amounts proportional to acne severity [62]. As a result, the use of TNF-α inhibitors has been explored as a potential treatment for severe and refractory cases of acne [63]. Some reports have suggested that TNF-α inhibitors may have a role in the management of acne fulminans [64,65]. In a 3-year retrospective data collection, a total of five different TNF-α inhibitors were tested, with adalimumab being the most commonly used [66]. Indeed, anti-TNF-α treatment may provide a rapid improvement in patients with acne fulminans when initial treatment with conventional therapies, isotretinoin, and prednisolone, has failed [66]. Hidradenitis suppurativa (HS), also known as acne inversa, is characterized by the formation of painful bumps, abscesses, and tunnels under the skin, primarily in those areas where skin rubs together, such as the armpits, groin, buttocks, or under the breasts: it typically begins after puberty and can persist for years [67]. A long-lasting inflammation, characterized by a large inflow of key-proinflammatory mediators such as TNF-α, IFN-γ, IL-1, IL-17, and IL-12/23, leads to the formation of scar tissues, which can result in skin changes and limited mobility of the affected areas [67]. The exact cause of HS is not fully understood, but it is believed to involve, also in this case, a combination of genetic, environmental, and immunity-related factors [68]. Based on both preclinical and clinical data, the TNF-α and CD4+ Th17 pathways may have relevant activities. Firstly, TNF-α supports Th17 polarization, increasing the ratio of Th17 to T-reg cells, which results in increased production of cytokines [69]. Secondly, TNF-α suppresses the adipocyte secretion of adiponectin, an anti-inflammatory hormone that regulates glucose metabolism and insulin sensitivity. Adiponectin levels are significantly decreased in HS patients, who, accordingly, often have higher fasting serum glucose and insulin levels or insulin resistance [70,71]. Thirdly, the relationship between smoking and HS might also involve TNF-α: nicotine increases eccrine gland secretion, and its presence in sweat induces keratinocytes and Th17 cells to release TNF-α. Moreover, nicotine directly stimulates macrophages to produce IL-1β and TNF-α [72]. Fourthly, TNF-α increases the expression of TLRs and MMPs [68,73,74]. The human anti-TNF-α monoclonal antibody adalimumab is currently the only biologic approved by both the US Food and Drug Administration and the European Medicines Agency for adults and adolescents with HS; however, a satisfactory clinical response has been only reported in approximately 50% of patients during phase III trials, suggesting the need for future further confirmation studies [75,76].

## 3. TNF-α after Exposition to Ultraviolet Light 

The skin is provided with the capability of counteracting a host of environmental stressors, including solar radiation, maintaining or restoring cutaneous homeostasis if disrupted [77]. These functions are coordinated by the cutaneous neuroendocrine system in synergy with the production of different biological factors induced by ultraviolet (UV) radiations, such as cytokines, biogenic amines, neuropeptides including pituitary and hypothalamic hormones as well as enkephalins, glucocorticoids, mineralocorticoids, and endocannabinoids [78,79]. This neurohormonal traffic regulates physiological skin functions separately or in concert, while disturbances in their activity may lead to disorders of inflammatory nature (i.e., psoriasis), hyperproliferative lesions, autoimmune diseases (i.e., vitiligo), premature aging, and even malignancies [80]. There is evidence that UV radiation induces early TNF-α release from keratinocytes [81]. Upregulation of TNF-α by UVB irradiation represents an important component of the inflammatory cascade on the skin. More in detail, UVB irradiation induces TNF-α expression in both keratinocytes and dermal fibroblasts, with TNF-α mRNA induction seen as early as 1.5 h after UVB but not after UVA exposure [81]. Different cytokines in combination with UVB have specific effects on chemokine production by UV-irradiated keratinocytes. In particular, IL-1α, a cytokine present in the irradiated skin, substantially and synergistically enhances the induction of TNF-α by UVB through increased TNF-α gene transcription [81]. Moreover, UVB induces the activity of nitric oxide synthase in human dermal endothelial cells through a TNF-α-dependent pathway. These cells secrete additional cytokines that form a positive feedback loop upregulating TNF-α and downstream TNF-α-induced chemokines [82,83].

### Skin Aging

Deterioration of skin quality is a natural phenomenon occurring with increasing age due to the synergistic effects of chronological aging, photoaging, estrogen deficiency, and environmental factors such as exposition to UV radiation [84,85]. TNF-α is implicated in all aging processes of skin, and chronic inflammation mediated by TNF-α is thought to contribute to the formation of wrinkles and other signs of aging, including loss of body mass, poor hydration, disintegration of dermis and epidermis junctions [86]. Aged skin is characterized by a decrease in both collagen content and skin thickness, which results in dry and wrinkled skin that may easily become bruised or require a long time to heal [87]. In particular, TNF-α is capable of modulating the expression of the matrix metalloproteinase (MMP) gene and is responsible for inducing the production of MMP-9, a collagenase that triggers skin damage and does not allow its total repair. The transcription of MMP genes is regulated by the transcription factors AP-1 (activator protein-1) and nuclear factor-kappa B (NF-κB) [88]. More precisely, TNF-α increases the binding activity of these transcription factors to the MMP-9 DNA sequence, stimulating MMP-9 production, which brings about collagen degradation [88]. There are more than 30 different kinds of collagen documented: collagen type I is normally seen in association with type III collagen in the skin. However, changes in collagen distribution during wound healing and extracellular matrix remodeling may alter the ratio of collagen I and collagen III, with higher amounts of collagen III during healing and increased collagen I in a healed wound [89]. Moreover, TNF- α can directly reduce type I collagen gene expression [90].

**Figure 4:**
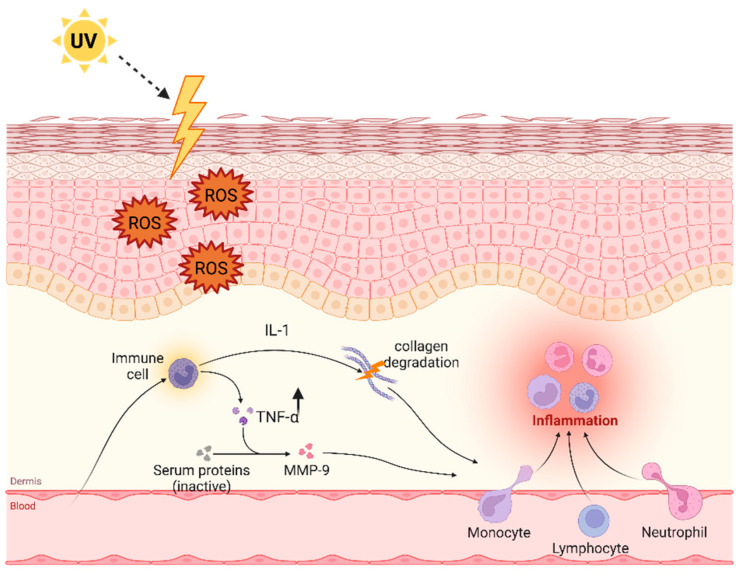
The role of tumor necrosis factor-alpha (TNF-α) in the process of skin aging (with ultraviolet radiation being implicated in the aging process). Reactive oxygen species (ROS) are generated by ultraviolet radiations and other factors. In direct or indirect ways, ROS can activate various intracellular kinases, leading to the production of TNF-α, which stimulates the production of matrix metalloprotease (MMPs). This event causes the reduction of collagen type I and III.

As mentioned above, macrophages that reside in the skin secrete TNF-α, but macrophages in the skin of older people secrete only a very small amount of this cytokine: this results in a defective activation of dermal blood vessels and, hence, a decreased recruitment of antigen-specific CD4+T cells within areas of the skin where antigens would have entered [91]. Isolated cutaneous macrophages from older people can be induced to secrete a notable amount of TNF-α after Toll-like receptor (TLR1, 2 or 4) ligand stimulation: this shows that macrophages residing in old skin are not defective but could be inactivated via CD4+Fox3+ regulatory T cells [92] (see Figure 4).

## 4. Discussion

A variety of biological functions can be attributed to TNF-α, and the intimate mechanisms of its activities appear not yet fully understood. Indeed, TNF-α, conferring resistance to certain types of infections on the one hand and causing pathological complications on the other, carries out contradictory roles. However, it has become clear that TNF-α displays an outstanding position in the defense against viral, bacterial, and parasitic infections and also in the incitement of autoimmune responses [1]. It also has a position in the pathogenesis of different autoinflammatory disorders, which result from an abnormal expression of innate immunity that variably affects several organs, including the skin. In addition, when TNF-α represents the major player in the inflammatory responses of these conditions, targeting the main driver of inflammation may be useful to treat such patients in a personalized fashion [93,94]. Even so, a natural induction of TNF-α may be protective in the individual patient, but its overproduction may be harmful and even lethal [95]. The inflammatory properties of TNF-α stem from its activation of the proinflammatory cytokines IL-1 and IL-6 and also of numerous transcription factors, most importantly NF-kB, which is directly involved in many skin-related inflammatory networks [96]. As seen in this review, a dysregulated activity of TNF-α can contribute to the pathogenesis of various skin disorders (see Table 1 for a list of the biological effects of TNF-α on different skin diseases): understanding its functions has led to the development of therapeutic strategies that might target TNF-α to manage at least a portion of inflammatory, autoimmune and autoinflammatory diseases seeing in TNF-α a pathogenic bedrock.

It is widely accepted that disrupted activity of the inflammasome, a large intracellular multiprotein platform with a central role in innate immunity, can lead to the overproduction of proinflammatory cytokines, such as IL-1β and TNF-α, and to a pathological delay in the modulation of inflammation firing in different organs and tissues, including the skin [97,98]. TNF inhibitors have demonstrated efficacy in lessening skin cell turnover and improving symptoms in many patients with heterogenous inflammatory conditions [99]. TNFR-associated periodic syndrome (TRAPS) is the most common autosomal-dominant autoinflammatory disease, caused by mutations in the *TNFRSF1A* gene, characterized by recurrent attacks of fever and variable inflammatory phenotypes, also involving the skin: the identification of *TNFRSF1A* mutations as the genetic cause of TRAPS coincided with the wider use of biological agents in medicine and raised the possibility that blocking TNF could potentially represent the primary therapeutic goal in TRAPS patients [100]. Another autoinflammatory disorder with important skin manifestations is cryopyrin-associated periodic syndrome (CAPS), characterized by recurrent episodes of systemic inflammatory attacks in the absence of recognized infections or proven autoreactive manifestations [101]: a young boy with a severe form of CAPS was treated with etanercept, a TNF-α blocker consisting of a fusion protein fusing the TNFR to IgG_1_ antibody, obtaining a dramatic improvement of his joint symptoms, but not of his skin disease [102]. Another potentially life-threatening complication of different infections and rheumatologic disorders affecting the skin, which originates from a massive hyper-cytokinemia, particularly IL-1, IL-6, IL-18, IFN-γ and TNF-α, is macrophage activation syndrome: many studies are in progress with the aim of monitoring the disease severity using blood cytokine signatures and improving anti-cytokine treatment of this condition [103]. Kawasaki disease is a further autoinflammatory disease primarily involving young children, with substantial risk of coronary artery involvement, which may receive benefit from the use of human-murine chimeric monoclonal anti-TNF-α antibody infliximab in the case of non-responsiveness to the conventional therapies [104]. However, all these interventions must be carefully established case by case and monitorized due to the multifaceted roles of TNF-α in skin pathology. 

Although the efficacy of anti-TNF agents has been established for several inflammatory diseases, a portion of these patients do not respond adequately, and this might be only partially attributed to pharmacokinetics, while doses needed to achieve locally sufficient drug concentrations can also induce immunosuppression and lead to secondary effects [105]. In the last decade, the success of all injectable therapies against TNF-α or anti-TNF-based immunotherapies has been more than remarkable; however, some questions remain concerning the long-term effectiveness of these drugs, mostly in consideration of side effects, their general tolerance, and eventual resistance shown by some patients [106]. In addition, some natural products that exhibit an inhibitory effect on TNF-α, blocking the formation of the ‘active’ TNF trimer, are being identified using in silico methods to reduce TNF-induced cytotoxicity and avoid any adverse effects [107]. Therefore, it is important to focus further research on new therapeutic approaches, developing the next generation of therapies against TNF-α for longer-term use, hopefully free from the risk of side effects in patients with acute and chronic skin inflammatory diseases. 

In conclusion, TNF-α can be considered an ally for its pivotal role in the immunologic defense of skin, e.g., in the context of wound healing for recruiting immune cells to the wound site, inducing cell death, and removing damaged or infected cells, but also an enemy for its active involvement in the pathogenesis of different inflammatory sceneries at the skin level, contributing to disease progression if excessively produced and even leading to permanent sequelae.

## Figures and Tables

**Table 1:** Biologic effects of tumor necrosis factor-alpha on different skin diseases.

Skin Disease	TNF-Alpha Effect
Psoriasis	- ↑ inflammatory response - ↑ ICAM-1, VCAM-1, E-selectin and P-selectin - Keratinocyte activation - ↑ IL-23, IL-12, IL-17, IL-22 and IFN-gamma - Activation of macrophages and dendritic cells
Vitiligo	Melanogenesis alteration - ↓ MITF and MSH-R function - ↓ MC1-R mRNA expression - ↑ ROS Cytokine imbalance - ↑ ICAM-1 - ↑ cytotoxic Ly → IFN-gamma
Cutaneous lupus erythematosus	- B cell activation → ↑ IL-1, IL-6, IL-18 and IFN-gamma - Keratinocyte apoptosis - Autoantibody production
Acne vulgaris and inversa	- Th17 polarization - ↓ adipocyte secretion of adiponectin - ↑ expression of TLRs and MMPs

ICAM-1: intercellular adhesion molecule-1; VCAM-1: vascular cell adhesion molecule; IL: interleukin; IFN-gamma: interferon-gamma; MITF: microphthalmia-associated transcription factor; MSH-R melanocyte-stimulating hormone receptor; MC1-R: melanocortin-1 receptor; ROS: reactive oxygen species; Ly: lymphocyte; TLRs: Toll-like receptors; MMPs: matrix metalloproteinases.

## Data Availability

Data sharing is not applicable to this article, as no datasets were generated or analyzed during this present study.

## Abbreviations

TNF-α: Tumor necrosis factor alpha; TNFR1: Tumor necrosis factor alpha receptor 1; TNFR2: Tumor necrosis factor alpha receptor 2; TRADD: TNFR1-associated death domain protein; FADD: FAS-associated death domain protein; RIP1: Receptor interacting protein 1; NIK: Nuclear factor-κB inducing kinase; MEK: serine/tyrosine/threonine kinase; MAPK: Mitogen-activated protein kinase; NF-κB: nuclear factor kappa-light-chain-enhancer of activated B cells; IKK: inhibitor of nuclear factor-κB kinase; JNK: c-Jun N-terminal kinase; AP-1: Activator protein 1; IFN-γ: Interferon-gamma; CCL20: Chemokine (C-C motif) ligand 20; UV: ultraviolet light; ROS: reactive oxygen species; MMP: matrix metalloprotease.
